# Rethinking False Positive Exercise Electrocardiographic Stress Tests by Assessing Coronary Microvascular Function

**DOI:** 10.1016/j.jacc.2023.10.034

**Published:** 2024-01-16

**Authors:** Aish Sinha, Utkarsh Dutta, Ozan M. Demir, Kalpa De Silva, Howard Ellis, Samuel Belford, Mark Ogden, Matthew Li Kam Wa, Holly P. Morgan, Ajay M. Shah, Amedeo Chiribiri, Andrew J. Webb, Michael Marber, Haseeb Rahman, Divaka Perera

**Affiliations:** British Heart Foundation Center of Excellence and National Institute for Health Research Biomedical Research Center at the School of Cardiovascular Medicine and Sciences, King’s College London, London, United Kingdom

**Keywords:** acetylcholine flow reserve, coronary flow reserve, coronary microvascular dysfunction, coronary physiological assessment, exercise stress test, false positive rate

## Abstract

**Background:**

Exercise electrocardiographic stress testing (EST) has historically been validated against the demonstration of obstructive coronary artery disease. However, myocardial ischemia can occur because of coronary microvascular dysfunction (CMD) in the absence of obstructive coronary artery disease.

**Objectives:**

The aim of this study was to assess the specificity of EST to detect an ischemic substrate against the reference standard of coronary endothelium-independent and endothelium-dependent microvascular function in patients with angina with nonobstructive coronary arteries (ANOCA).

**Methods:**

Patients with ANOCA underwent invasive coronary physiological assessment using adenosine and acetylcholine. CMD was defined as impaired endothelium-independent and/or endothelium-dependent function. EST was performed using a standard Bruce treadmill protocol, with ischemia defined as the appearance of ≥0.1-mV ST-segment depression 80 ms from the J-point on electrocardiography. The study was powered to detect specificity of ≥91%.

**Results:**

A total of 102 patients were enrolled (65% women, mean age 60 ± 8 years). Thirty-two patients developed ischemia (ischemic group) during EST, whereas 70 patients did not (nonischemic group); both groups were phenotypically similar. Ischemia during EST was 100% specific for CMD. Acetylcholine flow reserve was the strongest predictor of ischemia during exercise. Using endothelium-independent and endothelium-dependent microvascular dysfunction as the reference standard, the false positive rate of EST dropped to 0%.

**Conclusions:**

In patients with ANOCA, ischemia on EST was highly specific of an underlying ischemic substrate. These findings challenge the traditional belief that EST has a high false positive rate.

Exercise electrocardiographic stress testing (EST) represents a ubiquitous, noninvasive, and low-cost functional test for the evaluation of patients with new-onset angina. However, its use has declined over the past decade because of the higher sensitivity of other noninvasive stress imaging modalities and the perceived high false positive rate of EST. In view of this, EST has been downgraded to a Class 2b recommendation in the latest European Society of Cardiology guidelines.^1^ It is important to remember that the accuracy of EST has historically been assessed and validated against its ability to detect the presence of obstructive coronary artery disease (CAD), with the reference standard being visual diameter stenosis on coronary angiography. However, we now know that myocardial ischemia can, and indeed in nearly one-third of cases does, occur in the absence of obstructive CAD due to coronary microvascular dysfunction (CMD).^2^ Therefore, it is conceivable that historical false positive EST results were due not to the poor specificity of EST as a diagnostic test but rather to the limitations of obstructive CAD as a reference standard for myocardial ischemia. The aim of this study was to examine the specificity of EST in detecting an ischemic substrate compared against the robust reference standard of coronary endothelium-independent and endothelium-dependent microvascular function in patients with angina and nonobstructive coronary arteries (ANOCA).

## Methods

### Study population

**Figure 1:**
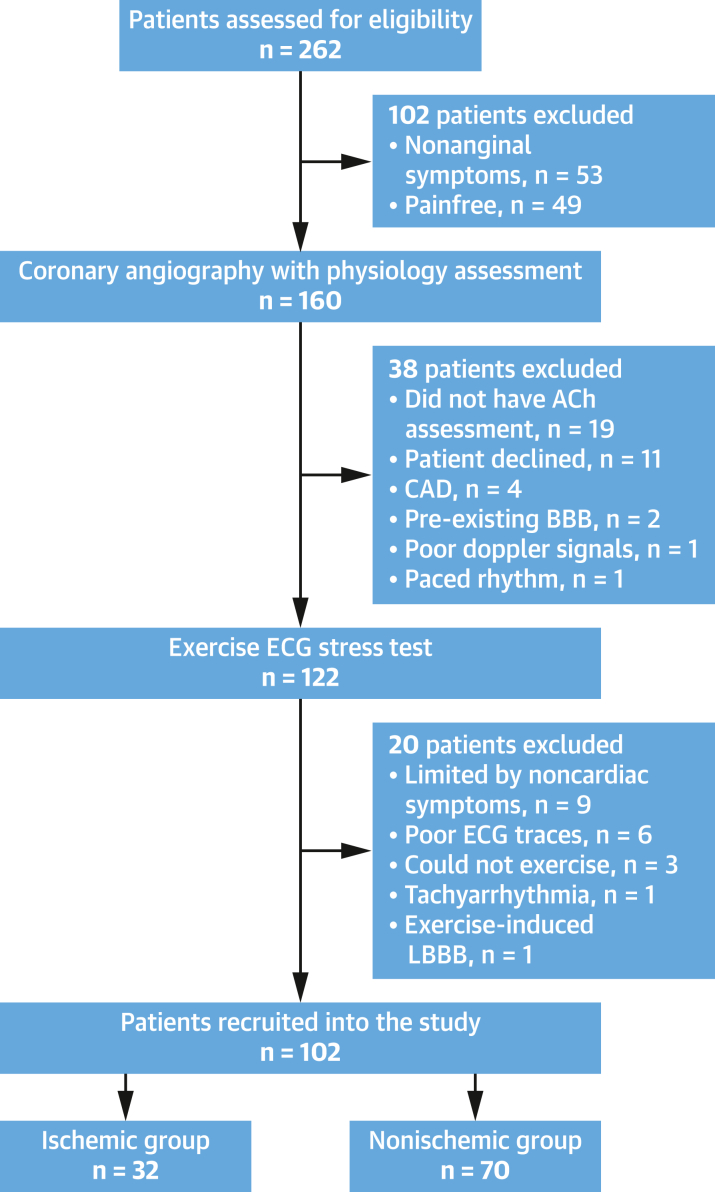
Consolidated Standards of Reporting Trials Diagram Demonstrating Study Flow This diagram demonstrates the number of patients assessed for eligibility and the reasons for exclusion. Overall, 262 patients with stable angina were assessed for eligibility, of whom 102 were excluded because of nonanginal symptoms or absence of symptoms. A total of 160 patients underwent coronary angiography with physiological assessment, of whom 38 were excluded. A total of 122 patients with comprehensive coronary physiological assessment (in response to both adenosine and acetylcholine [ACh]) underwent the mandated exercise ECG stress test, of whom 102 were included in the final analysis. BBB = bundle branch block; CAD = coronary artery disease; LBBB = left bundle branch block.

We prospectively enrolled consecutive patients presenting with angina who were referred for further assessment (Figure 1). Inclusion criteria were ANOCA (fractional flow reserve >0.80) and preserved left ventricular ejection fraction (>50%). Exclusion criteria were inability to undergo adenosine or acetylcholine assessment, chronic kidney disease (estimated glomerular filtration rate <30 mL/min/m^2^), significant valvular disease, history of acute coronary syndrome, previous revascularization, cardiomyopathy, limitation by nonanginal symptoms, existing bundle branch block, poor electrocardiographic (ECG) traces during exercise, and paced rhythm hindering ECG interpretation. All patients provided written informed consent in accordance with the protocol, which was approved by the UK National Research Ethics Service (20/LO/1294).

### Intracoronary physiological assessment

Our protocol for systematic evaluation of patients with ANOCA has been described in full previously.^3^ Briefly, all coronary physiological measurements were made in the left anterior descending coronary artery. A 0.014-inch dual sensor–tipped intracoronary guidewire was used for the measurement of distal coronary pressure and average peak flow velocity (APV). Aortic pressure was measured using the fluid-filled guide catheter. All patients received 1 mg intravenous midazolam, 200 μg intracoronary glyceryl trinitrate, and 70 U/kg unfractionated heparin prior to angiography and physiological assessment. We first assessed endothelium-independent microvascular function using intravenous adenosine (140 μg/kg/min), followed by endothelium-dependent microvascular function using graded intracoronary infusions of acetylcholine (18 μg/mL acetylcholine solution delivered at 1 mL/min followed by 2 mL/min) via the guide catheter. All intracoronary acetylcholine measurements were made at least 15 minutes after the intracoronary nitrate injection. Patients, researchers, and physiologists were blinded to the results of the coronary physiological assessment.

### Physiological data analysis

**Figure 2:**
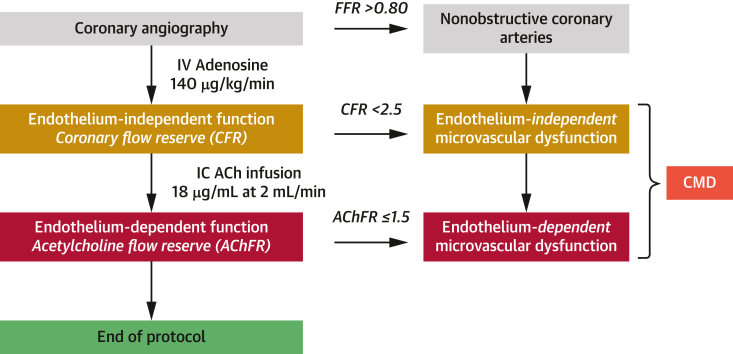
Coronary Physiological Assessment Protocol This is our standard clinical protocol that is used in all patients with angina and nonobstructive coronary arteries to identify an ischemic substrate. All patients undergo coronary angiography followed by intravenous (IV) adenosine assessment. Only patients with fractional flow reserve (FFR) >0.80 were included in this study. Coronary flow reserve (CFR) was calculated as the ratio of hyperemic average peak velocity (APV) in response to adenosine (140 μg/kg/min) and resting APV, with a value of <2.5 used to diagnose endothelium-independent microvascular dysfunction. Patients then underwent assessment with intracoronary (IC) acetylcholine (ACh) infusion (18 μg/mL), and ACh flow reserve (AChFR) was calculated as the ratio of volumetric coronary blood flow (CBF) during ACh infusion and CBF during rest. Volumetric CBF, in turn, was calculated as 0.5 × APV × cross-sectional area 5 mm distal to the Doppler sensor. AChFR ≤1.5 was diagnostic of endothelium-dependent microvascular dysfunction. Patients with CFR <2.5 and/or AChFR ≤1.5 were defined as having coronary microvascular dysfunction (CMD).

Signals were sampled at 200 Hz, with data exported into a custom-made study manager program (Academic Medical Center, University of Amsterdam) and analyzed using custom-made software (Cardiac Waves, King’s College London). Coronary flow reserve (CFR) was derived as adenosine-mediated hyperemic APV/basal APV; endothelium-independent microvascular dysfunction was defined as CFR <2.5.^3^^,^^4^ Hyperemic (minimal) microvascular resistance (hMR) was calculated as distal coronary pressure/APV during hyperemia. Elevated hMR was defined as hMR ≥2.5 mm Hg/cm/s. Acetylcholine flow reserve (AChFR) was calculated as the ratio of coronary blood flow (CBF) in response to acetylcholine infusion compared with basal CBF; endothelium-dependent microvascular dysfunction was defined as AChFR ≤1.5.^5^^,^^6^ The estimation of volumetric flow from Doppler flow velocity also incorporates vessel diameter. Given that acetylcholine can cause either epicardial vasodilation or vasoconstriction, volumetric CBF was calculated as quantitative coronary angiography–derived cross-sectional area × APV × 0.5, with quantitative coronary angiography performed 5 mm distal to the tip of the guidewire. CMD was defined as endothelium-independent and/or endothelium-dependent microvascular dysfunction (ie, CFR <2.5 and/or AChFR ≤1.5).^3^ Figure 2 describes our coronary physiological assessment protocol in patients with ANOCA.

### EST protocol

EST was performed after coronary angiography with physiological assessment. EST was performed using a Marquette Case 8000 system (GE Medical Systems) according to the American College of Cardiology and American Heart Association practice guidelines using a standard Bruce protocol.^7^^,^^8^ A 12-lead electrocardiogram, heart rate, and blood pressure were recorded at regular intervals before, during, and after EST. All exercise stress tests were supervised by cardiac physiologists who were blinded to the coronary physiological measurements. The only criterion for termination of the test was patient request.

Exercise time was defined as the time from the start of the exercise protocol to exercise cessation. Exercise-induced angina was documented when the patient reported chest tightness during exercise. Ischemic ECG changes were defined as the appearance of ≥0.1-mV horizontal or down-sloping ST-segment depression 80 ms from the J-point during exercise. All ECG tracings were reviewed by 3 independent observers blinded to the coronary physiological data; ischemic changes were adjudicated per the majority interpretation. Patients who developed ischemic ECG changes were classified as the “ischemic” group and those who did not as the “nonischemic” group. Patients were not requested to halt any medications prior to EST, which is representative of real-world practice.

### Statistical analyses

Normality of data was assessed using the Kolmogorov-Smirnov test. Normally distributed continuous data are presented as mean ± SD and were compared using the independent-samples Student’s *t*-test. Continuous data without normal distribution are presented as median (Q1-Q3) and were compared using the Mann-Whitney *U* test. Categorical variables are presented as number (%) and were compared using the chi-square test. Sensitivity, specificity, positive predictive value, and negative predictive value were determined for the development of ischemic ECG changes and exercise-induced angina against different reference standards of ANOCA with: 1) endothelium-independent microvascular dysfunction (CFR <2.5); 2) endothelium-dependent microvascular dysfunction (AChFR ≤1.5); and 3) endothelium-independent and/or endothelium-dependent microvascular dysfunction (ie, CMD; CFR <2.5 and/or AChFR ≤1.5). Values are presented as percentages. An apparent false positive rate was calculated as apparent false positives divided by the sum of apparent false positives and true negatives for the aforementioned reference standards. Apparent false positive rates were compared using the McNemar test. Binary logistic regression was performed using univariate analysis, and all statistically significant variables were entered into a multivariate model; ischemia on EST was the binary endpoint, and data are presented as OR (95% CI). Interobserver reliability in interpreting the exercise electrocardiogram for presence or absence of ischemia was assessed using the intraclass correlation coefficient. All analyses were performed using SPSS Statistics version 27 (IBM) and Prism version 9.0 for Windows (GraphPad Software).

### Sample size calculation

In a previous study, ischemic ECG changes on EST had 80% specificity in detecting coronary vasomotor dysfunction.^9^ Assuming a 30% rate of ischemic ECG changes on EST, we calculated that a sample size of 100 patients would give an absolute precision of 0.1 (95% CI) for a specificity of 91%.^10^

## Results

Between March 2021 and July 2023, 262 patients with stable angina were assessed for eligibility. Of these, 160 underwent coronary angiography with physiological assessment. A total of 122 patients with ANOCA underwent both adenosine and acetylcholine assessment in the catheterization laboratory and were deemed suitable to enroll into the study, of whom 102 were included in the final analysis (Figure 1). Twelve patients (12%) did not have any prior coronary-based investigations, 50 (52%) had prior coronary computed tomographic angiography scan, 8 (8%) had previous stress imaging (stress echocardiography or stress perfusion cardiac magnetic resonance imaging), and 27 (28%) had previous coronary angiography. EST took place 29 days (Q1-Q3: 20-139 days) after coronary angiography with physiological assessment. Thirty-two patients developed ischemic ECG changes during EST (ischemic group), whereas 70 did not (nonischemic group). There were no differences in gender, age, body mass index, cardiovascular risk factors, Canadian Cardiovascular Society angina grade, and NYHA functional class between the 2 groups (Table 1). Patients in the ischemic group had a higher percentage of typical angina (91% vs 73%; *P* = 0.043) and lower hemoglobin levels (130 ± 12 g/L vs 137 ± 14 g/L; *P* = 0.008) than those in the nonischemic group (Table 1). There were no differences in epicardial coronary physiology metrics (mean fractional flow reserve >0.90 in both groups), exercise time (348 ± 164 seconds vs 342 ± 164 seconds; *P* = 0.860), or the presence of exercise-induced angina during EST between the ischemic and nonischemic groups (Table 2).

**Table 1:** Baseline Characteristics

	Ischemic Group (n = 32)	Nonischemic Group (n = 70)	*P* Value
Patient demographics			
Female	18 (56)	48 (69)	0.227
Age, y	62 ± 6	59 ± 9	0.131
BMI, kg/m^2^	30 (25-32)	29 (25-34)	0.600
Hypertension	20 (63)	31 (44)	0.088
Diabetes mellitus	8 (25)	15 (21)	0.689
Hyperlipidemia	18 (56)	41 (59)	0.826
Smoking history	7 (22)	12 (17)	0.569
Symptomology			
Typicality score			
Nonanginal	0 (0)	0 (0)	
Atypical	3 (9)	19 (27)	0.043
Typical	29 (91)	51 (73)	
CCS grade			
I	3 (9)	6 (9)	
II	8 (25)	31 (44)	0.266
III	20 (63)	30 (43)	
IV	1 (3)	3 (4)	
NYHA functional class			
I	17 (53)	34 (48)	
II	14 (44)	30 (43)	0.593
III	1 (3)	6 (9)	
IV	0 (0)	0 (0)	
Laboratory results			
Hemoglobin, g/L	130 ± 12	137 ± 14	0.008
eGFR, mL/min/1.72 m^2^	79 ± 23	82 ± 18	0.555
NT-proBNP, pg/mL	74 (50-119)	65 (50-108)	0.540
HbA_1c_, mmol/mol	38 (36-43)	40 (38-42)	0.230
Total cholesterol, mmol/L	4.3 ± 1.2	4.2 ± 1.0	0.724
Medications			
Antiplatelet agents	18 (56)	33 (47)	0.393
Statins	23 (72)	48 (69)	0.736
ACEIs or ARBs	14 (44)	21 (30)	0.175
Beta-blockers	4 (13)	7 (10)	0.960
CCBs	1 (3)	6 (9)	0.231

Values are n (%), mean ± SD, or median (Q1-Q3).

ACEI = angiotensin-converting enzyme inhibitor; ARB = angiotensin receptor blocker; BMI = body mass index; CCB = calcium-channel blocker; CCS = Canadian Cardiovascular Society; eGFR = estimated glomerular filtration rate, NT-proBNP = N-terminal pro–brain natriuretic peptide; HbA_1c_ = glycated hemoglobin.

**Table 2:** Coronary Physiology and Exercise Electrocardiographic Stress Test Parameters

	Ischemic Group (n = 32)	Nonischemic Group (n = 70)	*P* Value
Coronary anatomy and physiology			
Diameter stenosis			0.128
<30%	28 (88)	67 (96)	
30%-50%	4 (12)	3 (4)	
Pd/Pa	0.95 ± 0.03	0.95 ± 0.03	0.807
FFR	0.90 ± 0.06	0.90 ± 0.04	0.971
CFR	2.3 (2.0-2.9)	2.6 (2.0-2.9)	0.507
hMR, mm Hg/cm/s	2.0 ± 0.8	2.1 ± 0.7	0.372
AChFR	1.2 ± 0.3	1.5 ± 0.6	<0.001
Endothelium-independent microvascular dysfunction (CFR <2.5)	20 (63)	30 (43)	0.066
Endothelium-dependent microvascular dysfunction (AChFR ≤1.5)	31 (97)	39 (56)	<0.001
CMD	32 (100)	46 (66)	<0.001
Exercise stress testing			
Exercise time, s	348 ± 164	342 ± 164	0.860
Presence of angina during EST	25 (78)	42 (60)	0.074
Peak heart rate, beats/min	145 ± 15	136 ± 23	0.035
Peak systolic blood pressure, mm Hg	191 ± 26	180 ± 34	0.137
Peak rate-pressure product, mm Hg × beats/min	27,662 ± 4,846	24,678 ± 7,130	0.039

Values are n (%), mean ± SD, or median (Q1-Q3).

AChFR = acetylcholine flow reserve; CFR = coronary flow reserve; CMD = coronary microvascular dysfunction; EST = exercise electrocardiographic stress testing; FFR = fractional flow reserve; hMR = hyperemic microvascular resistance.

All patients in the ischemic group had CMD, compared with 66% of patients in the nonischemic group (*P* < 0.001). There were no differences in CFR or hMR between the 2 groups; however, patients in the ischemic group had lower AChFR (1.2 ± 0.3 vs 1.5 ± 0.6; *P* < 0.001), as well as higher peak heart rate (145 ± 15 beats/min vs 136 ± 23 beats/min; *P* = 0.015) and rate-pressure product (27,662 ± 4,846 mm Hg × beats/min vs 24,678 ± 7,130 mm Hg × beats/min; *P* = 0.039) during exercise (Table 2). Sixty-three percent of patients in the ischemic group had impaired CFR compared with 43% of patients in the nonischemic group (*P* = 0.066); in contrast, 97% of patients in the ischemic group had impaired AChFR compared with 56% of patients in the nonischemic group (*P* < 0.001) (Table 2).

Using binary logistic regression analysis, AChFR, peak heart rate, and hemoglobin levels were independently associated with ischemic ECG changes during exercise (Table 3). Coronary endothelium-dependent microvascular dysfunction, but not coronary endothelium-independent microvascular dysfunction (both CFR <2.5 and CFR <2.0 thresholds), was associated with ischemic ECG changes during exercise (Supplemental Table 1). Elevated hMR alone was not associated with ischemic ECG changes during exercise (Supplemental Table 1).

**Table 3:** Predictors of Ischemia on Exercise Electrocardiographic Stress Testing

	OR (95% CI)	*P* Value
Univariate		
Age	1.045 (0.991-1.101)	0.103
Sex	0.589 (0.249-1.395)	0.229
Hypertension	2.097 (0.890-4.941)	0.090
Hemoglobin	0.956 (0.923-0.990)	0.012
Peak heart rate	1.025 (1.001-1.049)	0.039
Peak systolic blood pressure	1.010 (0.997-1.024)	0.138
CFR	0.979 (0.920-1.042)	0.506
AChFR	0.854 (0.776-0.939)	0.001
hMR	0.980 (0.924-1.039)	0.498
Multivariate (*R*^2^ = 0.228)		
AChFR	0.817 (0.720-0.928)	0.002
Hemoglobin	0.935 (0.894-0.979)	0.004
Peak heart rate	1.035 (1.006-1.064)	0.018

The units of increase in independent variables are as follows: age, 1 year older; hemoglobin, 1 g/L higher; peak heart rate, 1 beat/min higher; peak systolic blood pressure, 1 mm Hg higher; CFR, 0.1 unit higher; AChFR, 0.1 unit higher; and hMR, 0.1 mm Hg/cm/s higher.

Abbreviations as in Table 2.

Ischemic ECG changes during EST had poor sensitivity and moderate specificity to detect endothelium-independent microvascular dysfunction and a poor sensitivity but excellent specificity to detect endothelium-dependent microvascular dysfunction (Table 4). If obstructive CAD was used as the reference standard, then the assumed false positive rate of EST would be 31% (as all 102 patients had nonobstructive coronary arteries, but 32 patients had positive results on EST). With the addition of endothelium-independent microvascular dysfunction (ie, CFR <2.5) to the reference standard, the apparent false positive rate remained high at 23%. With the further addition of endothelium-dependent microvascular dysfunction (ie, AChFR ≤ 1.5) to the reference standard, the apparent false positive rate came down to 0% (*P* = 0.002 for false positive rate with endothelium-independent microvascular dysfunction vs endothelium-dependent microvascular dysfunction as the reference standard).

**Table 4:** Diagnostic Accuracy of Ischemia During Exercise Electrocardiographic Stress Testing to Detect Coronary Microvascular Dysfunction

	Endothelium-Independent Microvascular Dysfunction (CFR <2.5)	Endothelium-Dependent Microvascular Dysfunction (AChFR ≤1.5)	CMD (CFR <2.5 and/or AChFR ≤1.5)
Sensitivity	40	44	41
Specificity	77	97	100
PPV	63	97	100
NPV	57	44	34

Values are %.

NPV = negative predictive value; PPV = positive predictive value; other abbreviations as in Table 2.

Ischemic ECG changes during EST had poor sensitivity and moderate specificity to detect CFR < 2.0 or hMR ≥2.5 mm Hg/cm/s (Supplemental Table 2). Exercise-induced angina during EST had an excellent positive predictive value but poor negative predictive value to detect CMD (Supplemental Table 3). The composite of ischemic ECG changes and/or exercise-induced angina during EST had excellent sensitivity and positive predictive value but poor specificity and negative predictive value for detecting CMD (Supplemental Table 4).

There was a strong degree of interobserver reliability when interpreting exercise electrocardiograms for the presence or absence of myocardial ischemia (intraclass correlation coefficient = 0.843; 95% CI: 0.778-0.891).

## Discussion

The main findings of our study are as follows: 1) ischemia during EST had 100% specificity for detecting CMD in patients with ANOCA (Central Illustration); 2) patients who developed ischemia during exercise had lower AChFR; and 3) AChFR (or endothelium-dependent microvascular dysfunction) was the strongest predictor of ischemia during exercise.

**Central Illustration:**
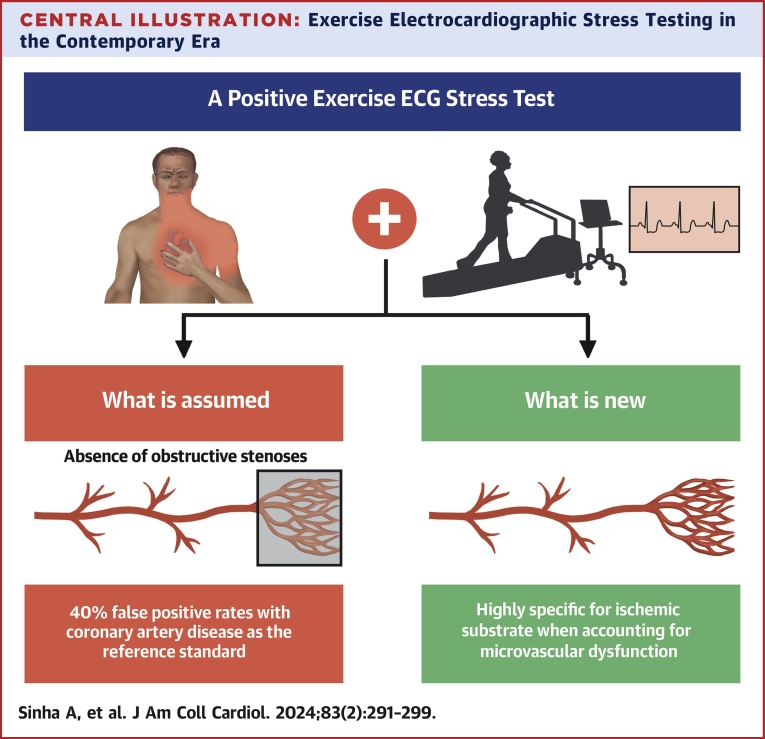
Exercise Electrocardiographic Stress Testing in the Contemporary Era Assessing the specificity of exercise electrocardiographic (ECG) stress testing against the contemporary gold standard of comprehensive coronary physiological assessment.

In recent years, there has been a paradigm shift in our understanding of ischemic heart disease, with the emphasis moving away from detecting obstructive CAD to confirming a physiological substrate for myocardial ischemia in the setting of chronic coronary disease. However, the diagnostic accuracy of traditional noninvasive tests has not been systematically re-evaluated against contemporary standards of assessing ischemia. Our study showed that the specificity and positive predictive value of EST are much higher when assessed against comprehensive physiological evaluation of the coronary circulation, in contrast to validation against the frequency of obstructive epicardial CAD.

### Specificity of EST for detecting an ischemic substrate

Previous studies that have examined the diagnostic accuracy of EST for the detection of ischemic substrate in patients with ANOCA (largely defined by an impaired CFR) have reported sensitivity values of 38% to 54%.^6^^,^^9^^,^^11^, ^12^, ^13^ Our study also showed that EST has poor sensitivity, but in contrast to previous studies,^6^^,^^11^, ^12^, ^13^ we found it to have excellent specificity to detect an underlying ischemic substrate. The reasons for this are likely 2-fold. First, previous studies have included angina (in the absence of ischemic ECG changes) as a criterion for a positive EST result. Our study demonstrates that angina revealed during EST is in fact poorly specific for an underlying ischemic substrate. Therefore, the use of angina as a criterion for a positive EST result may have led to under-reporting of specificity in previous studies. Second, several studies have used reference standards that do not interrogate the endothelium-dependent compartment of microvascular function, such as elevated index of microvascular resistance (>25),^11^^,^^13^ CFR <2 on positron emission tomography,^12^ and angiographic vasoconstriction in response to acetylcholine.^6^ Cassar et al^9^ used similar reference standards to our study and reported specificity of 80% of EST to detect an ischemic substrate. However, it is noteworthy that the symptomology of their patient cohort was unknown, along with delays of up to 6 months between EST and invasive physiological assessment.

### Physiological relevance of acetylcholine

Only AChFR, hemoglobin levels, and peak heart rate were associated with ischemic ECG changes during exercise, with AChFR and hemoglobin being lower and peak heart rate being higher in the ischemic group. This is suggestive that a combination of attenuated CBF and heightened myocardial oxygen demand was the underlying pathophysiology leading to ischemic ECG changes during exercise. There were no baseline demographic differences between the ischemic and nonischemic groups to account for the difference in peak heart rate between the 2 groups; it is noteworthy that although hemoglobin levels were associated with ischemic ECG changes during exercise, the mean hemoglobin levels were well within the normal limits in both ischemic and nonischemic groups. Finally, although peak heart rate and hemoglobin levels can be expected to be associated with ischemic ECG changes during exercise, ours is the first study to identify AChFR (and endothelium-dependent microvascular dysfunction) as being the strongest predictor of ischemic ECG changes during exercise. This reiterates the physiological relevance of acetylcholine testing in the evaluation of patients with ANOCA. Although adenosine acts on the A2A receptors on vascular smooth muscle cells to promote cyclic adenosine monophosphate-mediated vasodilatation, acetylcholine acts on endothelial muscarinic receptors, leading to cyclic guanosine monophosphate–mediated vasodilatation.^14^ Therefore, by assessing the response to adenosine, CFR reflects the theoretical (supra)maximal vasodilatory capacity of the vessel and may not be as physiologically relevant as AChFR. The latter is likely to be a better surrogate for the physiological flow-mediated vasodilatation that occurs during exercise, as it assesses the functionality of both the endothelial and vascular smooth muscle pathways (nitric oxide-cGMP-protein kinase G pathway). It is therefore unsurprising that AChFR, rather than CFR, was associated with ischemic ECG changes during EST.

Previous studies have demonstrated the safety and low complication rate of acetylcholine testing in the catheterization laboratory,^15^^,^^16^ while others have demonstrated the prognostic significance of endothelium-dependent microvascular dysfunction.^17^ Our findings may not only strengthen recommendations for intracoronary acetylcholine testing in future guidelines but also have implications from a therapeutic viewpoint, given the pleiotropic effects of statins and angiotensin-converting enzyme inhibitors on endothelial function and their prognostic benefit in patients with CMD.^18^

### Clinical implications

Coronary physiological assessment detects the substrate for myocardial ischemia (CFR <2.5 and/or AChFR ≤1.5), which are sensitive markers, as they detect perturbations early in the ischemic cascade. EST, in contrast, detects myocardial ischemia and is a specific marker. It is therefore unsurprising that all patients who developed ischemia during EST had an identifiable ischemic substrate in the catheterization laboratory but not vice versa. Our results show that a positive result on EST is highly specific for (and suggestive of) the presence of an ischemic substrate, but as this does not distinguish the relative contributions of the epicardial and microvascular compartments, EST will always have to be combined with a test that specifically evaluates the epicardial coronary arteries, namely, invasive or noninvasive coronary angiography. The pathway that is being increasingly adopted worldwide is to use coronary computed tomographic angiography as the first line investigation for patients presenting with chest pain that might be consistent with inducible ischemia. In this setting, patients who are found to have unobstructed epicardial arteries are managed in 1 of 3 ways: discharge without further investigation (a common strategy), consideration for a noninvasive functional test (adopted by networks in which there is good awareness of microvascular dysfunction), or referral for invasive testing (usually reserved for patients with a high burden of symptoms despite several antianginal medications). In this context, EST may have a role as a second-line test with good rule-in utility. This is likely to expedite the diagnosis of CMD in a large proportion of patients and streamline the use of (less widely available and more costly) tests, such as invasive physiology and/or stress perfusion cardiac magnetic resonance imaging. The efficacy of this proposed strategy would need to be tested in a future diagnostic trial. The more historical pathway (and one that is decreasingly recommended in international guidelines because of the perceived false positive rate) is to use EST as a first-line investigation. In this scenario, patients with positive results on EST would often go on to undergo invasive angiography to exclude epicardial disease. The findings of our study suggest that invasive microvascular physiological assessment may not be required if no obstructive coronary disease is found in these cases, as the positive EST result makes a diagnosis of CMD highly likely. Finally, longitudinal EST may play an important role in monitoring response to therapy in patients who have been diagnosed with CMD on the basis of a positive EST result.

### Study limitations

Our study had some limitations that should be considered when interpreting the findings. First, this was a single-center study with a relatively small sample size (although our study was adequately powered for our primary hypothesis). Our findings need confirmation in larger multicenter studies.

Second, all patients in this study had angina as their main symptom that necessitated further investigations. Therefore, these results may not necessarily apply to patients without angina (such as those with breathlessness as their predominant symptom).

Third, long-term outcome data are currently unavailable for these patients, precluding us from identifying features on EST that enable the risk stratification of patients; however, this will form the basis for future studies.

## Conclusions

Using comprehensive coronary physiology as the reference standard, ischemic ECG changes during exercise were highly specific for coronary microvascular dysfunction in our patient cohort. This is an important finding that highlights the limitations of using obstructive CAD as a reference standard to assess the accuracy of noninvasive imaging modalities.

Perspectives **COMPETENCY IN MEDICAL KNOWLEDGE:** Ischemic ECG changes during treadmill exercise stress testing have 100% specificity and positive predictive value for the detection of abnormal coronary microvascular function, indicating that the pattern should not be considered falsely positive.

**TRANSLATIONAL OUTLOOK:** Treadmill exercise stress testing is a widely available, cost-effective method to identify CMD in patients with angina and confirmed nonobstructive coronary arteries.

## Funding Support and Author Disclosures

This work is supported by grants from the Medical Research Council (MR/T029390/1), the British Heart Foundation (FS/16/49/32320), and the UK National Institute for Health Research (through the Biomedical Research Center award to King’s College London and Guy’s and St. Thomas’ Hospital). Prof Shah is supported by the British Heart Foundation (CH/1999001/11735). The authors have reported that they have no relationships relevant to the contents of this paper to disclose.
