# Supplementary material for: Rethinking False Positive Exercise Electrocardiographic Stress Tests by Assessing Coronary Microvascular Function
Source: J Am Coll Cardiol. 2024 Jan 16;83(2):291–9. doi: 10.1016/j.jacc.2023.10.034 (PMC10790243; doi:10.1016/j.jacc.2023.10.034)
Supplement: Supplemental Tables 1-4 [file mmc1.docx]

**Rethinking False Positive Exercise Electrocardiographic Stress Tests by Assessing Coronary Microvascular Function**

**Supplemental materials**

**Table of content**

[**Supplemental Table 1.** Predictors of ischemia (binary variables) during exercise ECG stress testing. 2](#_Toc147431018)

[**Supplemental Table 2.** Diagnostic accuracy of ischemia during exercise ECG stress testing to detect coronary flow reserve <2.0 and hyperemic microvascular resistance≥2.5mmHg.cm^-1^.s^-1^. 3](#_Toc147431019)

[**Supplemental Table 3.** Diagnostic accuracy of exercise-induced angina during exercise ECG stress testing to detect coronary microvascular dysfunction. 4](#_Toc147431020)

[**Supplemental Table 4.** Diagnostic accuracy of ischemia and/or exercise-induced angina during exercise ECG stress testing to detect coronary microvascular dysfunction. 5](#_Toc147431021)

**Supplemental Table 1.** Predictors of ischemia (binary variables) during exercise ECG stress testing.

|  | Odds ratio (95% CI) | P value |
| --- | --- | --- |
| Univariate | | |
| CFR<2.5 | 2.222 (0.942, 5.241) | 0.068 |
| CFR<2.0 | 0.963 (0.368, 2.523) | 0.939 |
| AChFR≤1.5 | **24.641 (3.184, 190.723)** | **0.002** |
| hMR≥2.5 | 1.357 (0.519, 3.547) | 0.534 |
| Multivariate (R^2^=0.184) | | |
| CFR<2.5 | 1.305 (0.507, 3.358) | 0.581 |
| AChFR≤1.5 | **22.570 (2.854, 178.463)** | **0.003** |

CFR: coronary flow reserve; AChFR: acetylcholine flow reserve; hMR: hyperemic microvascular resistance

**Supplemental Table 2.** Diagnostic accuracy of ischemia during exercise ECG stress testing to detect coronary flow reserve <2.0 and hyperemic microvascular resistance≥2.5mmHg.cm^-1^.s^-1^.

|  | **CFR<2.0** | **hMR≥2.5mmHg.cm^-1^.s^-1^** |
| --- | --- | --- |
| **Sensitivity** | 31% | 38% |
| **Specificity** | 68% | 69% |
| **PPV** | 25% | 28% |
| **NPV** | 74% | 78% |

PPV: positive predictive value; NPV: negative predictive value

**Supplemental Table 3.** Diagnostic accuracy of exercise-induced angina during exercise ECG stress testing to detect coronary microvascular dysfunction.

|  | **Endothelium-*independent* microvascular dysfunction**  (CFR < 2.5) | **Endothelium-*dependent* microvascular dysfunction**  (AChFR < 1.5) | **CMD**  (CFR<2.5 and/or AChFR≤1.5) |
| --- | --- | --- | --- |
| **Sensitivity** | 74% | 76% | 73% |
| **Specificity** | 42% | 58% | 58% |
| **PPV** | 55% | 81% | 85% |
| **NPV** | 63% | 51% | 40% |

PPV: positive predictive value; NPV: negative predictive value

**Supplemental Table 4.** Diagnostic accuracy of ischemia and/or exercise-induced angina during exercise ECG stress testing to detect coronary microvascular dysfunction.

|  | **Endothelium-*independent* microvascular dysfunction**  (CFR < 2.5) | **Endothelium-*dependent* microvascular dysfunction**  (AChFR < 1.5) | **CMD**  (CFR<2.5 and/or AChFR≤1.5) |
| --- | --- | --- | --- |
| **Sensitivity** | 84% | 86% | 82% |
| **Specificity** | 38% | 58% | 58% |
| **PPV** | 57% | 82% | 86% |
| **NPV** | 71% | 64% | 50% |

PPV: positive predictive value; NPV: negative predictive value
